# IFNα enhances the production of IL-6 by human neutrophils activated *via* TLR8

**DOI:** 10.1038/srep19674

**Published:** 2016-01-21

**Authors:** Maili Zimmermann, Fabio Arruda-Silva, Francisco Bianchetto-Aguilera, Giulia Finotti, Federica Calzetti, Patrizia Scapini, Claudio Lunardi, Marco A. Cassatella, Nicola Tamassia

**Affiliations:** 1 Department of Medicine, Division of General Pathology, University of Verona, Verona, Italy; 2 Department of Medicine, University of Verona, Verona, Italy

## Abstract

Recently, we reported that human neutrophils produce biologically active amounts of IL-6 when incubated with agonists activating TLR8, a receptor recognizing viral single strand RNA. In this study, we demonstrate that IFNα, a cytokine that modulates the early innate immune responses toward viral and bacterial infections, potently enhances the production of IL-6 in neutrophils stimulated with R848, a TLR8 agonist. We also show that such an effect is not caused by an IFNα-dependent induction of TLR7 and its consequent co-activation with TLR8 in response to R848, but, rather, it is substantially mediated by an increased production and release of endogenous TNFα. The latter cytokine, in an autocrine manner, leads to an augmented synthesis of the IkBζ co-activator and an enhanced recruitment of the C/EBPβ transcription factor to the *IL-6* promoter. Moreover, we show that neutrophils from SLE patients with active disease state, hence displaying an IFN-induced gene expression signature, produce increased amounts of both IL-6 and TNFα in response to R848 as compared to healthy donors. Altogether, data uncover novel effects that type I IFN exerts in TLR8-activated neutrophils, which therefore enlarge our knowledge on the various biological actions which type I IFN orchestrates during infectious and autoimmune diseases.

Neutrophils are the first and the most numerous innate immune cells recruited to the sites of infection, where they play a crucial role in destroying and eliminating invading pathogens^1^. Because of their powerful microbicidal equipment, neutrophils are often depicted as harmful cells that can cause damage to the surrounding tissues during acute inflammation^1^. Nonetheless, extensive research performed in the last decades has recognized neutrophils as highly versatile and sophisticated cells displaying an important role in linking the innate and adaptive arms of the immune response, as well as a significant synthetic capacity^2^,^3^. For instance, neutrophils produce and release a wide range of cytokines having pro-inflammatory, anti-inflammatory and immunoregulatory actions as a result of their interactions with microbes and other environmental substances^3^. Ligands for Toll-like receptors (TLR) or other pattern recognition receptors (PRR) function, in fact, as very powerful stimuli for cytokine expression in neutrophils^4^,^5^,^6^,^7^. In this context, we have recently reported that human neutrophils can *de novo* express and produce biologically active amounts of IL-6^8^; however, they do so only after an overnight incubation, and more significantly in response to engagement of TLR8– which commonly recognizes single strand RNA (ssRNA) of viral origin^9^. Accordingly, we have shown that the induction of IL-6 production by neutrophils primarily occurs because TLR8 agonists are able to trigger a sequence of time-dependent molecular events, which, ultimately, remodel the chromatin at the *IL-6* genomic locus from an “inactive” to an “active” configuration^8^. Most of these events were found to be sustained and amplified by endogenous TNFα (also produced in abundant amounts in response to TLR8 activation) and include, among others: the *de novo* expression of the co-activator IκBζ, which is required to drive IL-6 transcription^10^; the induction of a latent enhancer located 14 kb upstream of the *IL-6* transcriptional start site (TSS); the CCAAT/enhancer binding protein-β (C/EBPβ) recruitment to, as well as histone acetylation induction at, *IL-6* regulatory regions^8^. Notably, the identification of neutrophils as potential source of IL-6, as well as the molecular mechanisms specifically regulating such a function, has definitively clarified controversial literature in the field^11^. Moreover, in light of findings demonstrating that neutrophils produce IL-6 upon incubation with respiratory syncytial virus (a single-stranded RNA virus)^12^, recognize HIV-1 *vi*a TLR8^13^, and express a broad repertoire of functional PRR involved in the recognition of nucleic acids of viral origin^6^,^7^, our data have further corroborated the notion that neutrophils should be included among the cells responding to viral infections^14^.

Type I interferon (IFN) is known to mediate the early innate immune responses to viral infections, acting either directly, by inhibiting viral replication, or indirectly, by activating and potentiating effector functions exerted by immune cells^15^. Interestingly, type I IFN targets also human neutrophils, for instance by prolonging their survival^16^, or by inducing the expression of CXCL10 mRNA^17^, and the production of biologically active TNF-related apoptosis-inducing ligand (TRAIL)^18^. There is also evidence that type I IFN potentiates the expression of IL-6, for example in double strand RNA (dsRNA)-stimulated HeLa cells^19^, or in circulating PBMCs of chronic hepatitis C patients^20^. Besides, an uncontrolled production of type I IFN is involved in the pathogenesis of autoimmune diseases, including systemic lupus erythematosus (SLE) or rheumatoid arthritis^21^.

Based on these premises, herein we investigated whether type I IFN modulates the production of IL-6 by TLR8-activated neutrophils and, if so, at which molecular level. To validate the biological significance of *in vitro* results, we also investigated whether neutrophils isolated from patients with active SLE, hence displaying the “IFN-signature”, produce altered levels of IL-6 in response to activation *via* TLR8.

## Results

### IFNα potentiates the production of IL-6 by human neutrophils incubated with R848

**Figure 1:**
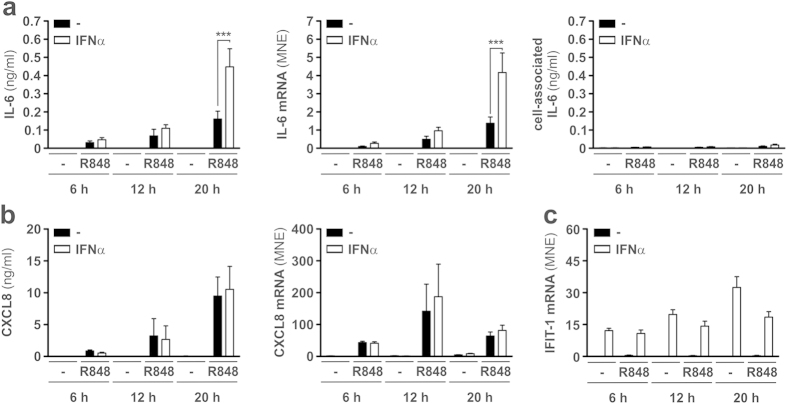
IFNα enhances the production of IL-6 in R848-treated neutrophils. Neutrophils (5 × 10^6^/ml), isolated from the peripheral blood of healthy donors, were cultured with or without 5 μM R848, 1000 U/ml IFNα or IFNα plus R848 for 6, 12 and 20 h to evaluate: released (**a**, left panel) and cell-associated (**a**, right panel) IL-6, as well as released CXCL8 (**b**, left panel), by ELISA; IL-6 (**a**, central panel), CXCL8 (**b**, right panel), and IFIT1 (**c**) mRNA expression, by RT-qPCR. ELISA values stand for the mean ± SEM (n = 3–11). Gene expression data (mean ± SEM, n = 3–9) are depicted as mean normalized expression (MNE) units after GAPDH mRNA normalization. Asterisks indicate a significant enhancement by IFNα: ***p < 0.001, by 2-way ANOVA followed by Bonferroni’s post-test.

To investigate the effect of type I IFN on the production of IL-6 by TLR8-stimulated neutrophils, we incubated highly purified neutrophils (99.7 ± 0.2 % purity)^22^ with or without 5 μM R848^8^, in the presence or absence of 1000 U/ml IFNα^18^, for up to 20 h. We found that IFNα, while not triggering *per se* any IL-6 production (Fig. 1a, left panel) or mRNA expression (Fig. 1a, middle panel), enhanced the yields of IL-6 recovered in supernatants from neutrophils incubated with R848, as well as the accumulation of the related mRNA (Fig. 1a, middle panel). Such a potentiation was significantly evident only after 20 h of cell incubation (Fig. 1a, left and middle panels), and not due to an enhanced secretion of an intracellularly stored pool of IL-6 (Fig. 1a, right panel). Under the same experimental conditions, IFNα neither triggered the expression of CXCL8, nor influenced the stimulatory effect of R848 on the CXCL8 production and mRNA accumulation (Fig. 1b), while it potently induced the accumulation of IFIT1 mRNA (Fig. 1c), a classical interferon-dependent gene^23^. Taken together, these data demonstrate that IFNα greatly enhances the production of IL-6 by neutrophils stimulated with R848.

### IFNα does not induce the expression of TLR7 in human neutrophils

**Figure 2:**
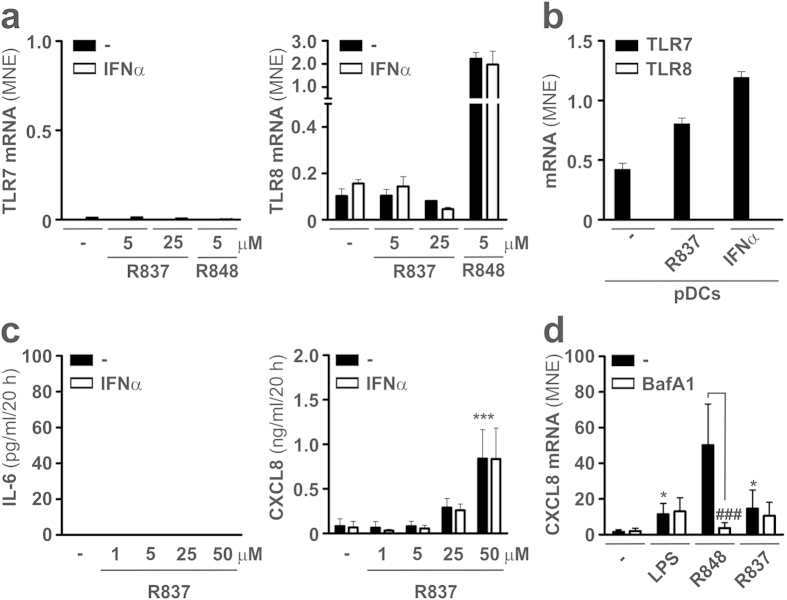
TLR7 is not expressed in human neutrophils incubated with IFNα. (**a**) TLR7 (left panel) and TLR8 (right panel) mRNA expression either in (**a**), neutrophils cultured with or without 5 μM R848, 5 and 25 μM R837, in the presence or absence of 1000 U/ml IFNα for 20 h, or (**b**), pDCs cultured for 5 h with or without 5 μM R837 or 1000 U/ml IFNα. In (**a**), no significant effect by IFNα was found by 2-way ANOVA followed by Bonferroni’s post-test. (**c**) IL-6 and CXCL8 levels detectable in supernatants from neutrophils treated for 20 h with or without 1–50 μM R837, in the presence or absence of 1000 U/ml IFNα. (**d**) CXCL8 mRNA expression in neutrophils pretreated with 25 nM Bafilomycin A1 (BafA1) for 30 min and then incubated for 5 h with 100 ng/ml LPS, 5 μM R848 or 50 μM R837. Values in panels (**a,c,d**) stand for the mean ± SEM (n = 5), while panel (**b**) depicts a representative experiment out of three independent ones with similar results. Asterisks in panel (**c,d**) indicate a significant increase with respect to untreated cells, while ^#^ symbols in panel (**d**) indicate a significant inhibition exerted by BafA1: *p < 0.05, *** and ^###^p < 0.001, by 2-way ANOVA followed by Bonferroni’s post-test.

To identify the mechanisms whereby IFNα augments the production of IL-6 in R848-treated neutrophils, we initially investigated whether an IFNα-mediated *de novo* induction of TLR7 and its consequent co-activation with TLR8 could occur. In this regard, we have recently shown that resting mature neutrophils do not express TLR7^8^. However, earlier observations have reported that SLE-serum treated neutrophils express TLR7 and respond to its specific ligand, R837^24^, by producing augmented levels of CXCL8^25^. Such an acquisition to express TLR7 mRNA and, in turn, respond to R837, was suggested to be caused by the presence of type I IFN in SLE serum, also because, *in vitro*, 1000 U/ml IFNα were shown to induce TLR7 mRNA expression in neutrophils from healthy donors^25^. In the same study, IRS-661 (a specific TLR7 inhibitor) was found to inhibit the upregulation of CD83 in plasmacytoid dendritic cells (pDCs) treated with supernatants harvested from SLE neutrophils previously incubated with anti-ribonucleoprotein antibodies, the latter being shown to trigger neutrophil extracellular trap release *via* TLR7^25^. In our populations of highly purified neutrophils incubated for 20 h with 1000 U/ml IFNα, either alone or in combination with either R848 or R837, no induction of TLR7 mRNA was, however, detected (Fig. 2a, left panel). Similarly, IFNα did not influence the expression of TLR8 mRNA (Fig. 2a, right panel), which, instead, was found to be remarkably upregulated by R848 (Fig. 2a, right panel). Control experiments confirmed^26^ that TLR7, but not TLR8, mRNA is expressed in human pDCs under resting conditions, TLR7 being upregulated upon pDC incubation with R837 or IFNα (Fig. 2b).

Consistent with the lack of TLR7 mRNA expression, no IL-6 production was observed in neutrophils stimulated for 20 h by up to 50 μM R837, either in the absence or in the presence of IFNα (Fig. 2c, left panel). On the other hand, we could confirm that R837 stimulates the production of CXCL8 by neutrophils (Fig. 2c, right panel), but only at elevated concentrations (e.g., 25–50 μM) and, once again, without being influenced by the IFNα co-addition (Fig. 2c, right panel). Such R837-induced CXCL8 production occurred in a TLR7-independent manner (Fig. 2d), as it was not abrogated by the pretreatment of neutrophils with BafilomycinA1 (BafA1), a potent inhibitor of endosomal acidification that is a required condition for efficient TLR7 and TLR8, but not TLR4, signaling^27^ (Fig. 2d). Altogether, data prove that the enhanced IL-6 production by neutrophils co-treated with IFNα and R848 is not mediated by the co-activation of TLR8 with newly induced TLR7. Data also indicate that neutrophil-derived CXCL8 in response to R837 is independent of endosomal TLR7 (as well as TLR8), and, likely, occurs *via* other, not yet identified mechanisms.

### Endogenous TNFα partially mediates the upregulatory effect of IFNα on the R848-stimulated IL-6 production

**Figure 3:**
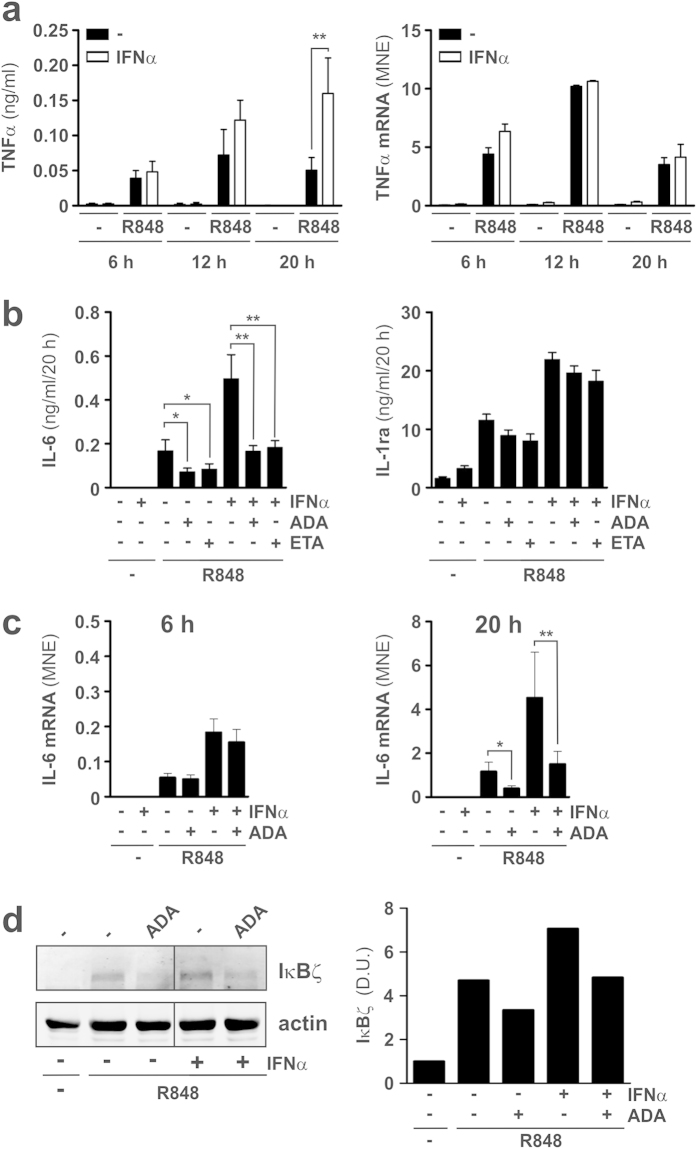
Role of endogenous TNFα in mediating the enhancing effect of IFNα on the production of IL-6 by R848-treated neutrophils. **(a)** Neutrophils (5 × 10^6^/ml), isolated from the peripheral blood of healthy donors, were cultured with or without 5 μM R848, 1000 U/ml IFNα or IFNα plus R848 for 6, 12 and 20 h to evaluate: released (n = 3–11) (left panel), TNFα, as measured by ELISA, and TNFα mRNA expression (right panel), by RT-qPCR (n = 3–8). Asterisks indicate significant increase: **p < 0.01, by 2-way ANOVA followed by Bonferroni’s post-test. **(b)** IL-6 (left panel) and IL-1ra (right panel) levels in supernatants from neutrophils pretreated for 30 min with or without 10 μg/ml ADA or 10 μg/ml ETA and then incubated for further 20 h with 1000 U/ml IFNα and/or 5 μM R848. Values represent the mean ± SEM (n = 5). **(c)** IL-6 mRNA expression in neutrophils pretreated for 30 min with or without 10 μg/ml ADA and then incubated for further 6 (left panel) or 20 h (right panel) with 1000 U/ml IFNα and/or 5 μM R848. Values represent the means ± SEM (n = 5). In (**b,c**), asterisks indicate significant inhibition by ADA: *p < 0.05, **p < 0.01, by 1-way ANOVA followed by Tukey’s post-test. (**d**) IκBζ antigen expression (by western blot analysis) in neutrophils pretreated for 30 min with or without 10 μg/ml ADA and then incubated for further 20 h with 1000 U/ml IFNα and/or 5 μM R848. Samples were run on the same gel, but lanes were noncontiguous. A representative experiment out of two independent ones with similar results is shown (left panel). The graph (right panel) illustrates the relative densitometric quantification of IκBζ levels (normalized by actin).

Additional experiments uncovered that also the production of TNFα is significantly augmented by IFNα in neutrophils incubated with R848 for 20 h (by approximately three-fold, Fig. 3a, left panel). Interestingly, no significant changes of the TNFα transcript accumulation were observed in IFNα plus R848-treated neutrophils as compared to cells treated with R848 only (Fig. 3a, right panel), indicating that IFNα enhances the production of TNFα likely by acting at the translational level. Therefore, at the light of our recently published observations, briefly described in the introduction^8^, we investigated the contribution of endogenous TNFα in mediating the enhancement of IL-6 expression in neutrophils treated with IFNα plus R848. To do so, we used two TNFα neutralizing drugs, namely adalimumab (ADA) and etanercept (ETA)^28^, and compared the grade of their inhibitory effects on the production of IL-6 by neutrophils incubated for 20 h with R848 only *versus* neutrophils incubated with IFNα and R848. As shown in left panel of Fig. 3b, the release of IL-6 by neutrophils treated with IFNα and R848 was inhibited to a slightly higher extent than in neutrophils treated with R848 only (61.1 ± 3.7 % *versus* 55.7 ± 2.1 in the case of ADA; by 58.4 ± 3.9 % *versus* 50.5 ± 2.0 % in the case of ETA, n = 5), indicating that endogenous TNFα is crucial for the IFNα-dependent IL-6 enhancement. Yet, neither ADA, nor ETA, reduced the production of IL-6 by neutrophils co-treated with IFNα and R848 to the levels detected in supernatants harvested from neutrophils treated with R848 only. Nonetheless, in IFNα plus R848-treated neutrophils, ADA reduced the accumulation of IL-6 transcripts only after 20, but not 6, h of incubation (Fig. 3c). Furthermore, that endogenous TNFα greatly contributes to mediate the upregulatory effect of IFNα on the production of IL-6 was further supported by the western blot experiment displayed in Fig. 3d. The latter, in fact, shows that ADA inhibits the enhanced expression of IκΒζ protein that is detectable in neutrophils treated with IFNα plus R848 as compared to cells treated with R848 only. Notably, results shown in right panel of Fig. 3b also show that TNFα neutralizing drugs do not suppress all cytokines produced by neutrophils treated with R848 and/or IFNα. In fact, blocking the activity of autocrine TNFα did not significantly influence the release of IL-1ra by neutrophils incubated with R848, IFNα, or IFNα plus R848, the latter combination triggering a synergistic IL-1ra production. All in all, data indicate that the increased production of TNFα occurring in neutrophils co-treated with IFNα and R848, with respect to neutrophils incubated with R848 only, largely mediates their enhanced production of IL-6.

### IFNα potentiates the R848-induced recruitment of C/EBPβ to the IL-6 genomic locus

**Figure 4:**
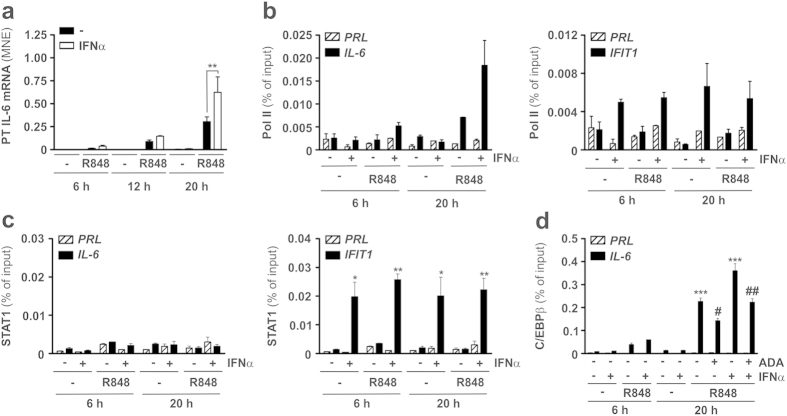
Effect of IFNα on Pol II, STAT1 and C/EBPβ recruitment to the IL-6 promoter in R848-treated neutrophils. **(a)** Neutrophils (5 × 10^6^/ml), isolated from the peripheral blood of healthy donors, were cultured with or without 5 μM R848, 1000 U/ml IFNα or IFNα plus R848 for 6, 12 and 20 h to evaluate IL-6 primary transcript (PT) expression by RT-qPCR (n = 3–8). Asterisks indicate a significant increase: **p < 0.01, by 2-way ANOVA followed by Bonferroni’s post-test. Evaluation of Pol II (**b**) and STAT1 (**c)** binding to the *IL-6* (**b,c**, left panel) or to the *IFIT1* (**b,c**, right panel) promoters by ChIP analysis in neutrophils incubated for 6 h and 20 h with or without 1000 U/ml IFNα and/or 5 μM R848. **(d)** Neutrophils were pretreated for 30 min with or without 10 μg/ml ADA and then incubated for further 6 or 20 h with 1000 U/ml IFNα and/or 5 μM R848 to be processed for ChIP analysis using C/EBPβ Abs. In panels **b–d**, the co-immunoprecipitated DNA samples were amplified using specific primer pairs and expressed as percent of the total input. Panel **b** depicts a representative experiment out of two ones with similar results while values in panels **c,d** stand for the mean ± SEM (n = 3). Asterisks in panel (**c,d**) indicate a significant increase with respect to untreated cells while ^#^ symbols indicate a significant inhibition exerted by ADA: * and ^#^p < 0.05, ** and ^##^p < 0.01 and ***p < 0.001, by 1-way ANOVA followed by Tukey’s post-test.

Results from primary transcript (PT) experiments (Fig. 4a), as well as from ChIP of Polymerase II (Pol II) recruitment to the *IL-6* TSS (Fig. 4b, left panel), indicated that the potentiation of IL-6 expression in neutrophils co-treated with IFNα and R848 for 20 h occurred at the transcriptional level. At the light of these data, we subsequently investigated whether IFNα, directly or indirectly, activates transcription factors (TFs) able to transactivate IL-6 gene expression, including C/EBPβ^29^. In this context, it has been recently shown that type II IFN/IFNγ, known to potently upregulate cytokine production in neutrophils^30^, also increases the transcription of IL-6 in human and murine macrophages stimulated with TLR ligands, *via* induction of a sustained signal transducer and activator of transcription 1 (STAT1) and IRF-1 occupancy at the *IL-6* locus^31^. However, no recruitment of either STAT1 (Fig. 4c) or IRF-1 **(data not shown**) to the promoter (Fig. 4c) or enhancers **(data not shown**) of the *IL-6* locus was detected in neutrophils treated with IFNα, either alone or in combination with R848. Conversely, an evident Pol II (Fig. 4b, right panel) and STAT1 (Fig. 4c, right panel) recruitment at the *IFIT1* promoter occurred in response to IFNα, at both the 6 and 20 h time-points. Instead, in neutrophils co-treated with IFNα plus R848 we detected an increased recruitment of C/EBPβ to the *IL-6* promoter with respect to neutrophils treated with R848 only (Fig. 4d), which, interestingly, was measured already after 6 h of incubation. Notably, the increased recruitment of C/EBPβ to the *IL-6* promoter of neutrophils treated for 20 h with IFNα plus R848 was partially, but not completely, reduced by ADA to the levels reached in neutrophils treated with R848 only, similarly to what observed in the case of IL-6 release (Fig. 3b) and IL-6 mRNA (Fig. 3c). Taken together, data demonstrate that, in R848-treated neutrophils, IFNα increases IL-6 transcription in a STAT1/IRF1-independent manner. Data also demonstrate that, under the same experimental conditions, IFNα augments the recruitment of C/EBPβ to the *IL-6* promoter induced by R848 in a manner partially dependent on endogenous TNFα.

### IFNα does not increase the prosurvival effect that R848 exerts on neutrophils

**Figure 5:**
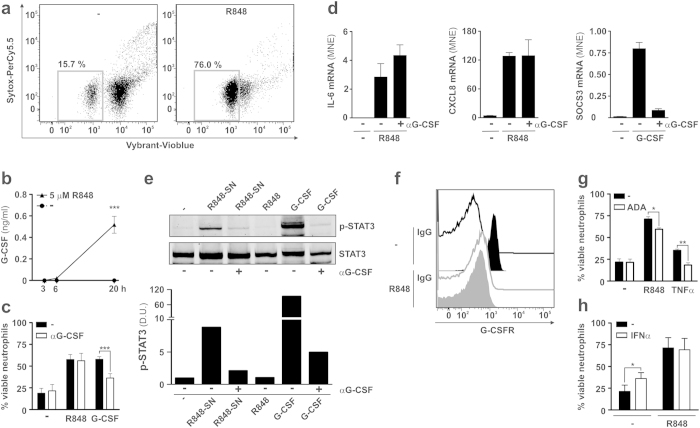
The enhanced viability of R848-treated neutrophils is partially dependent on endogenous TNFα, but not G-CSF. (**a**) Neutrophils were cultured with or without 5 μM R848 for up to 20 h, to be processed for viability evaluation by flow cytometry analysis. The percentage of alive cells was defined as Vybrant/Sytox double negative cell population (grey boxes). **(b**) G-CSF levels in supernatants from neutrophils cultured with or without 5 μM R848 for up to 20 h. (**c,g,h**), viability of neutrophils after culture for 20 h with 5 μM R848 (**c,g,h**), 1000 U/ml G-CSF (**c**), 10 ng/ml TNFα (**g**), in the presence or not of, respectively, 10 μg/ml mAbs neutralizing G-CSF (**c**), 10 μg/ml ADA (**g**), or 1000 U/ml IFNα (**h**). Data are expressed as mean ± SEM (n = 3 for panel **c**, n = 6 for panels **g** and **h**), while asterisks indicate a significant inhibition (for panels **c,g**) or increase (for panel **h**), as it follows: *p < 0.05, **p < 0.01, ***p < 0.001, by 2-way ANOVA followed by Bonferroni’s post-test. **(d)** IL-6, CXCL8 and SOCS3 mRNA expression in neutrophils pretreated for 30 min with or without 10 μg/ml neutralizing G-CSF mAbs and then incubated for further 20 h with 5 μM R848 or 1000 U/ml G-CSF. Error bars represent the SEM calculated from triplicate qPCR reactions. A representative experiment out of three independent ones with similar results is shown. **(e)** STAT3 tyrosine phosphorylation in freshly isolated neutrophils, either untreated or cultured for 60 min with 5 μM R848, 1000 U/ml G-CSF or supernatants from allogenic neutrophils incubated for 20 h with 5 μM R848 (R848-SN), in the presence or absence of 10 μg/ml neutralizing G-CSF mAbs (representative experiment, n = 2). The graph in the lower panel displays the relative densitometric quantification of p-STAT3 levels (normalized by total STAT3). **(f)** Neutrophils cultured for 20 h with or without 5 μM R848 were analysed by flow cytometry for G-CSFR membrane expression using an anti-G-CSFR (filled histogram) or matched isotype control (empty histogram) mAbs.

Given the well-known tendency of neutrophils to undergo apoptosis once cultured *in vitro*^32^, the observation that both IL-6 and TNFα are produced at maximal levels after an overnight incubation with IFNα plus R848 might appear intriguing. It should be, however, mentioned that TLR8 ligands have been already shown to delay neutrophil apoptosis^33^,^34^,^35^, as also confirmed by our flow cytometric analysis by Vybrant DyeCycle violet/Sytox stain (Fig. 5a). Because also IFNα delays neutrophil apoptosis^36^,^37^, we next investigated whether potential factors whereby R848 delays neutrophil apoptosis also favor the production of IL-6, and whether IFNα had a positive effect on them, to ultimately enhance IL-6 expression. Since, R848-treated neutrophils produce, in addition to TNFα, also high amounts of G-CSF (Fig. 5b), and given that both G-CSF and TNFα delay neutrophil apoptosis^32^,^38^, we initially asked whether endogenous G-CSF and/or TNFα could play a role in mediating the prosurvival effect of R848. In the case of G-CSF, we found that G-CSF-blocking antibodies did not change the viability of neutrophils observed after 20 h of culture in the presence of R848 (Fig. 5c), even though they significantly decreased the prosurvival effect of exogenous G-CSF (Fig. 5c). Consistently, G-CSF neutralization did not have any effect on the induced IL-6 or CXCL8 mRNA accumulation in R848-treated neutrophils (Fig. 5d), while it almost completely abolished the induction of SOCS3 mRNA in response to exogenous G-CSF (Fig. 5d). Notably, neutrophil-derived G-CSF was biologically active, as supernatants harvested from R848-activated neutrophils (R848-SN in Fig. 5e) triggered, in 60 min, a G-CSF-dependent STAT3 tyrosine phosphorylation in freshly isolated heterologous neutrophils (Fig. 5e). Interestingly, the inability of R848-treated neutrophils to respond to endogenous G-CSF was found to likely rely on a complete downregulation of G-CSFR (Fig. 5f). Conversely, ADA, as well as ETA (**data not shown**), significantly decreased the prosurvival effect exerted by R848 in neutrophils (by 25 ± 4% at 20 h, n = 6) (Fig. 5g). Nonetheless, despite of the fact that it potently upregulates the production of TNFα in R848-treated neutrophils (Fig. 3a), IFNα did not exert any additional prosurvival effect on top of that promoted by R848 alone (Fig. 5h). All in all, data demonstrate that R848-induced viability is partially dependent on endogenously produced TNFα. Data, however, exclude that IFNα amplifies the production of IL-6 by R848-treated neutrophils simply because it enhances neutrophil survival.

### R848-treated neutrophils do not express type I IFNs but produce increased levels of IL-6 when coincubated with type II IFN

**Figure 6:**
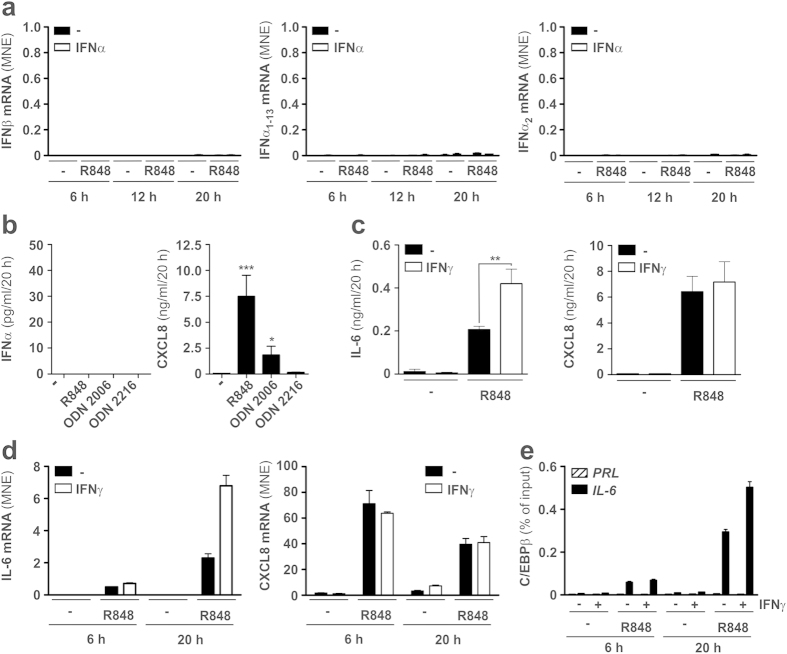
R848-treated neutrophils do not express type I IFN but produce higher levels of IL-6 when coincubated with IFNγ. (**a**) Neutrophils were cultured with or without 5 μM R848, 1000 U/ml IFNα or IFNα plus R848 for 6, 12 and 20 h to evaluate IFNβ, IFNα_1–13_ and IFNα_2_ mRNA expression by RT-qPCR (n = 3–8). In panel (**b**) neutrophils were cultured with or without 5 μ μM R848, 2 μM CpG ODN 2006 and 2 μM CpG ODN 2216 for 20 h and then IFNα (left panel) and CXCL8 (right panel) release were measured by specific ELISA. In panel (**c**–**e**) neutrophils were cultured with or without 5 μM R848, 100 U/ml IFNγ or IFNγ plus R848 for 20 h (**c**), or 6 and 20 h (**d,e**), to evaluate: i) IL-6 (**c**, left panel) and CXCL8 (**c**, right panel) production by ELISA; ii) IL-6 (**d**, left panel) and CXCL8 (**d**, right panel) mRNA expression by RT-qPCR; iii) C/EBPβ binding to the IL-6 and PRL promoters by ChIP analysis (**e**). Panels (**d**,**e**) depict a representative experiment out of two ones with similar results. ELISA values in panel (**b**,**c**) stand for the mean ± SEM (n = 5). Asterisks indicate a significant increase over control (**b**), or enhancement by IFNγ (**c**): *p < 0.05, **p < 0.01, ***p < 0.001, by 1-way ANOVA (**b**) or 2-way ANOVA (**c**).

Next, we ruled out any autocrine action of potential endogenous type I IFN in regulating IL-6 production by TLR8-activated neutrophils. RT-qPCR experiments, in fact, failed to detect any mRNA accumulation for both IFNβ (Fig. 6a, left panel) and IFNα, in the latter case as measured using primers recognizing all IFNα transcripts (IFNα_1–13_, Fig. 6a, central panel), or specifically IFNα_2_ (Fig. 6a, right panel). Lack of IFNα production was also confirmed at the protein level, as revealed by ELISA testing supernatants harvested from neutrophils treated for 20h, not only with R848, but also with various CpG preparations (CpG-ODN 2006 and 2216) (Fig. 6b, left panel), which are known to activate TLR9^39^. As expected^39^, pDCs incubated for 20 h with either CpG-ODN 2216, or R848, were found to release significant amounts of IFNα (**data not shown)**. On the other hand, neutrophils released significant amounts of CXCL8 in response to CpG-ODN 2006 and R848 (Fig. 6b, right panel), indicating that they were properly activated.

In other experiments, and supporting previous findings obtained in murine macrophages^31^, we observed that also type II IFN/IFNγ remarkably enhances the production of IL-6 by neutrophils incubated with R848. The latter phenomenon was observed to occur after 20 h of neutrophil incubation (Fig. 6c, left panel), concomitantly with an upregulation of IL-6 transcripts (Fig. 6d, left panel). Similarly to IFNα, while IFNγ did not exhibit any effect on CXCL8 mRNA expression (Fig. 6c, right panel) and protein production in R848-treated neutrophils (Fig. 6d, right panel), its effect on the increased IL-6 mRNA expression was accompanied by an enhanced C/EBPβ recruitment to the IL-6 promoter (Fig. 6e).

### Production of IL-6 in response to TLR7 and/or TLR8 agonists by SLE neutrophils

**Figure 7:**
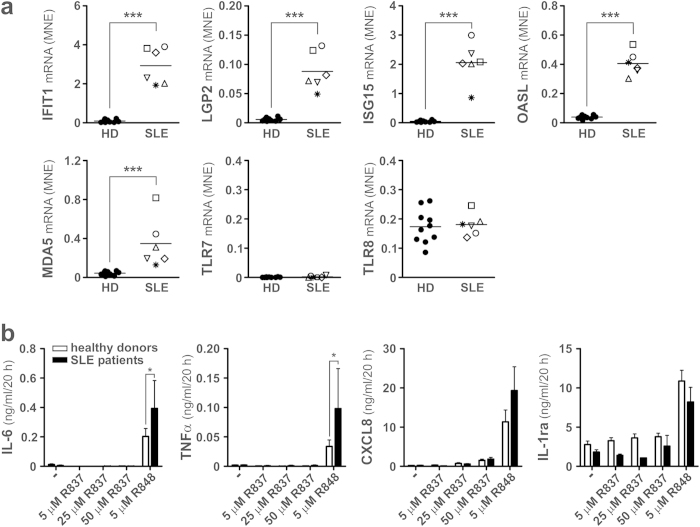
Gene expression profile and IL-6, TNFα, CXCL8 and IL-1ra production by neutrophils isolated from SLE patients. (**a**) IFIT1, LGP2, ISG15, OASL, MDA5, TLR7 and TLR8 mRNA expression in neutrophils freshly isolated from the peripheral blood of SLE patients with high SLEDAI (n = 6, each symbol identifying a different patient, see Table 1) and healthy donors (n = 10). Asterisks (***p < 0.001, by Student’s t test) indicate a significant difference between the two groups. (**b**) Amounts of IL-6, TNFα, CXCL8 and IL-1ra produced by peripheral neutrophils isolated from SLE patients (n = 6) and healthy donors (n = 6) cultured for 20 h in the presence of 5 μM R848 and 5–50 μM R837. Asterisks indicate a significant increase: *p < 0.05, by 2-way ANOVA followed by Bonferroni’s post-test.

In a final series of experiments, we investigated whether highly purified neutrophils from SLE patients with active disease (SLEDAI > 5, see Table 1 for patient characterization), thus constitutively displaying remarkably elevated levels of IFN-dependent genes, such as IFIT1, LGP2, IGS15, OASL and MDA5 (Fig. 7a), produce more IL-6 than neutrophils from healthy donors (HD) in response to TLR8 and/or TLR7 agonists. We found that SLE neutrophils treated with 5 μM R848 produced twice as much IL-6 than control neutrophils (Fig. 7b). SLE neutrophils also released significantly more TNFα in response to R848 (Fig. 7b), and, interestingly, showed a tendency to release more CXCL8 than HD neutrophils (Fig. 7b). However, no increase of IL-1ra could be observed under the same experimental conditions (Fig. 7b), in line with the proinflammatory status of these SLE neutrophils^40^. By contrast, SLE neutrophils neither produced IL-6 or TNFα, nor significantly upregulated their IL-1ra production in response to 5–50 μM R837 (Fig. 7b), in accordance with their lack of TLR7 mRNA expression (Fig. 7a). Nonetheless, CXCL8 was released in a dose-dependent manner by both control and SLE neutrophils, yet at similar levels (Fig. 7b). Finally, no IFNα expression/production was observed under R848 or R837 stimulation (**data not shown**). The fact that neutrophils isolated from patients with active SLE – thus constitutively displaying the so-called IFN signature (Fig. 7a) – produce higher levels of both IL-6 and TNFα in response to R848 than HD neutrophils is consistent with the *in vitro* effects of IFNα on the same cytokines (Figs 1a and 3a). It is also plausible that factors other than type I IFN might control the cytokine-producing capacity of neutrophils in SLE patients, as suggested by their different behavior to release CXCL8 and IL-1ra in response to R848 as compared to HD neutrophils incubated with IFNα and R848.

## Discussion

IL-6 is a multifunctional cytokine involved in regulation of the immune system. As a potent pro-inflammatory cytokine, IL-6 plays a pivotal role in host defense against pathogens and acute stress^41^. Nonetheless, IL-6 plays also a role in the pathogenesis of inflammatory and autoimmune diseases^41^. Recently, we demonstrated that upon activation of TLR8 by specific imidazoquinolines exerting antiviral activities, including R848 and CL075, neutrophils display the capacities to produce IL-6 in biologically active amounts^8^. Since neutrophils outnumber other immune cells under diverse inflammatory conditions, a detailed knowledge on how their production of IL-6 is regulated is of notable interest. In such regard, very crucial cytokines that, amongst others, modulate cytokine expression of immune cell and consequently also innate immune responses, are type I IFNs^15^. While being protective during acute viral infections, type I IFNs can also have deleterious roles in bacterial infections and autoimmune diseases^42^, including pathologies in which neutrophils are involved, such as sepsis^43^, pediatric SLE^25^ and rheumatoid arthritis^44^. In this study, we show that TLR8-activated neutrophils produce approximately three times more IL-6 when cultured for 20 h in the presence of IFNα than in its absence, a phenomenon controlled at the level of both mRNA transcription and accumulation. Similarly, we report that neutrophils isolated from SLE patients with active disease produce significantly higher levels of IL-6 than neutrophils from healthy donors, when stimulated *in vitro* with R848 for 20 h. Because neutrophils from SLE patients displayed a strong “IFN signature”, it is tempting to speculate that their increased capacity to produce IL-6 likely depends on previous *in vivo* exposures to circulating type I IFN, thus consistent with *in vitro* experiments. Instead, no direct effect of IFNα on IL-6 gene expression could be detected. Also type II IFN/IFNγ was found to remarkably enhance the production of IL-6 by neutrophils treated with R848, further highlighting the capacity of these cells to fully respond to the interferon-induced signals during viral and autoimmune diseases.

In the attempt to clarify the molecular bases of such an IFNα-dependent enhancement of neutrophil-derived IL-6 we could exclude that IFNα does so simply by increasing the viability of neutrophils. In fact, even though we confirmed that TLR8 activation potently prolongs the survival of neutrophils^12^,^33^,^34^,^35^, viability of neutrophils incubated in the presence of both IFNα and R848 did not differ from that measured in neutrophils incubated in the presence of R848 only. Notably, under the latter experimental conditions, neutrophils were found to release remarkable amounts of biologically active G-CSF, as demonstrated by the capacity of supernatants harvested from R848-treated neutrophils to trigger a G-CSF-dependent STAT3 phosphorylation in heterologous neutrophils. However, contrary to our expectations, endogenous G-CSF was ineffective in R848-treated neutrophils, as surface G-CSFRs were completely downregulated. The biological meaning of such a G-CSFR disappearance in R848-treated neutrophils is unknown, but it has been observed to occur also in LPS-, TNFα-, fMLF- and C5a-treated cells^45^ as well as *in vivo*, after intravenous injection of LPS^46^. By contrast, we uncovered that the extended survival of R848-treated neutrophils partially depends on endogenous TNFα. Whether the enhanced viability mediated by endogenous TNFα also helps to sustain IL-6 production in R848-treated neutrophils remains to be demonstrated.

We also excluded that the IFNα-dependent enhancement of neutrophil-derived IL-6 is caused by an induction of TLR7 and its consequent co-activation with TLR8 in response to R848. Under our experimental conditions, neither neutrophils from healthy donors incubated with IFNα, nor SLE neutrophils, were found to express TLR7 or respond to the TLR7-specific agonist R837 in terms of IL-6 production. These findings are in partial contradiction with the results of a previous publication reporting that neutrophils isolated from juvenile SLE patients express TLR7 mRNA, as well as that, *in vitro*, 1000 U/ml IFNα could induce TLR7 mRNA expression in neutrophils from HDs^25^. While it should be kept in mind that juvenile and adult SLE are two clinically distinct diseases^47^, in which circulating neutrophils may be likely exposed to different mediators and, eventually, function diversely, other factors might explain the differences between our results and those by Garcia-Romo and collegues^25^. For instance, knowing that PBMCs express discrete levels of TLR7^48^, it is plausible to hypothesize that potential contaminating PBMCs might have influenced the data outcome in the work by Garcia-Romo and collegues^25^; in fact, while neutrophils are isolated at a purity level greater than 99.7% in our hands^8^,^49^, in the study by Garcia-Romo and collegues^25^ neutrophils were stated greater than 98 % pure. On the same line, other studies, in which no precaution for completely removing all possible contaminating leukocytes were undertaken, have reported that resting neutrophils isolated from the blood could express low levels of TLR7 mRNA^33^,^50^,^51^. By contrast, no TLR7 mRNA expression was detected when neutrophils were isolated to high levels of purity, in particular in terms of contaminating eosinophils^52^,^53^. How critical is the purity of neutrophils to obtain genuine and reliable results in the context of gene expression studies has been already evidenced^3^,^54^.

Interestingly, we found that both neutrophils from healthy donors and SLE patients similarly respond to R837 in terms of CXCL8 production, but only if the TLR7 agonist was used at 25–50 μM. In this latter case, our observations confirm the results by Garcia-Romo *et al.*^25^, who also showed that juvenile SLE neutrophils produce CXCL8 in response to 36 μM R837^25^. However, since in our experiments CXCL8 produced by R837-treated neutrophils was not abrogated by the pretreatment of neutrophils with BafilomycinA1, it is our opinion that it likely occurs *via* other, not yet identified, TLR7-independent mechanism. Our observations are, in any case, consistent with previous findings demonstrating that chemotaxis and H_2_O_2_ release induced by R837 in human neutrophils occur in an IRAK4-independent manner, thus without activating a canonical, TLR-activated MyD88-dependent signaling pathway^55^. In this study, we also demonstrate that the augmentation of IL-6 production by IFNα in R848-treated neutrophils largely coincides with an enhanced production and release of TNFα, which, in turn, appears to substantially mediate it. Even neutrophils from SLE patients with active disease were found to produce greater TNFα amounts than healthy controls in response to R848, further supporting our *in vitro* results on the effects of IFNα on TNFα expression as well. Consistent with the role of endogenous TNFα in mediating the effects of IFNα on IL-6 gene expression, we demonstrated an enhanced recruitment of C/EBPβ to the IL-6 promoter in IFNα plus R848-treated neutrophils^8^. Although the notion that endogenous TNFα may mediate the enhanced IL-6 production exerted by IFNα in TLR8-activated neutrophils is not so surprising at the light of our recently published data^8^, it nonetheless emphasizes how important is TNFα for neutrophil physiopathology. This is further highlighted in studies showing that the interferon gene expression signature in neutrophils from rheumatoid arthritis patients correlates with a good response to anti-TNF therapy^44^, once again indicating that IFN activity is mediated *via* TNFα induction.

Concomitantly, we failed to detect any expression of type I IFN in neutrophils incubated with IFNα and/or R848, thus excluding an autocrine action by endogenous type I IFN in regulating the production of IL-6. In our hands, lack of IFNα production was also observed in neutrophils treated with CpG-ODN 2006 and 2216, namely under experimental conditions previously shown by Lindau and colleagues^56^ to induce neutrophil-derived IFNα at greater levels than R848. In the latter study^56^, neutrophils were stated to be approximately 99.8 % pure, thus excluding the presence of contaminating cells likely producing IFNα. Moreover, that our neutrophil preparations were properly activated was demonstrated by the fact that they released significant amounts of CXCL8 in response to CpG-ODN 2006. Therefore, we do not know how to explain why we failed to detect type I IFN in neutrophils. One possible explanation is that we might have found, by chance, “non-responder donors”, as also occurred to Lindau and colleagues themselves^56^. In summary, our data uncover that TLR8 ligands, IFNα and TNFα, three players often coexisting in many diseases of viral or autoimmune origin, promote a strong production of IL-6 in human neutrophils placing this cell type among potential targets for immunotherapeutic interventions.

## Materials and Methods

### Cell purification and culture

Granulocytes were isolated from buffy coats of healthy donors or, for selected experiments, from peripheral blood of SLE patients (see below) and as previously described, manipulated under endotoxin-free conditions^57^. Briefly, buffy coats or peripheral blood was centrifuged on Ficoll-Paque PLUS gradient (1078 g/ml density, GE Healthcare, Little Chalfont, United Kingdom) at 400 × *g* for 30 min, at room T, at a 1:1 ratio. Then, granulocytes were obtained by dextran sedimentation followed by hypotonic lysis of erythrocytes. Finally, neutrophils were isolated to reach 99.7 ± 0.2 % purity by positively removing all contaminating cells using the EasySep neutrophil enrichment kit (StemCell Technologies, Vancouver, Canada)^8^,^22^. Neutrophils were then suspended at 5 × 10^6^/ml in RPMI 1640 medium supplemented with 10 % low endotoxin FBS (<0.5 EU/ml; from BioWhittaker-Lonza, Basel, Switzerland), treated or not with 1000 U/ml pegylated IFNα-2a (Pegasys, Roche, Basel, Switzerland), 100 U/ml IFNγ (R&D Systems, Minneapolis, MN, USA) 5 μM R848, 5–50 μM R837, 2 μM CpG ODN 2006 and 2 μM CpG ODN 2216 (Invivogen, San Diego, CA, USA), 10 ng/ml TNFα (Peprotech, Rocky Hill, NJ, USA), 100 ng/ml ultrapure LPS from *E. coli* 0111:B4 strain (Alexis, Enzo Life Sciences, Farmingdale, NY, USA), 1000 U/ml G-CSF (Myelostim, Italfarmaco Spa, Milano, Italy), and then plated either in 6/24-well tissue culture plates or in polystyrene flasks (from Greiner Bio-One, Kremsmünster, Austria) for culture at 37°C, 5% CO_2_ atmosphere. In selected experiments, neutrophils were preincubated for 30 min with 10 μg/ml adalimumab (Humira, Abbott Biotechnology Limited, Barceloneta, Puerto Rico), 10 μg/ml etanercept (Enbrel, Amgen, Thousand Oaks, CA, USA), or 5 μg/ml anti-human G-CSF (Clone 3316, R&D Systems), prior to stimulation. After the desired incubation period, neutrophils were either processed for Chromatin Immunoprecipitation (ChIP) experiments, or collected and spun at 300 ×*g* for 5 min, for other assay types. In the latter case, cell-free supernatants were immediately frozen in liquid nitrogen and stored at −80 °C, while the corresponding cell pellets were either extracted for total RNA or lysed for protein analysis. pDCs were isolated as previously described^58^ using the BDCA-4 Diamond Isolation Kit (Miltenyi Biotec, Bergisch Gladbach, Germany).

### ELISA

Cytokine concentrations in cell-free supernatants and cell-lysates were measured by commercially available ELISA kits, specific for human IL-6 (eBioscience, San Diego, CA, USA), TNFα (eBioscience), G-CSF (R&D Systems), IL-1ra (R&D Systems), CXCL8 (Mabtech, Nacka Strand, Sweden) and IFNα (Mabtech). Detection limits of these ELISA were: 8 pg/ml for IL-6 and IFNα, 4 pg/ml for TNFα, 40 pg/ml for IL-1ra, 16 pg/ml for G-CSF and CXCL8.

### Flow cytometry

Phenotypic studies were performed exactly as previously described^8^. For G-CSFR staining, 10^5^ neutrophils, incubated with or without 5 μM R848 for the indicated times, were harvested and stained for 15 min at room T with 1:20 PE anti-human CD114 (G-CSF-R) mAb (clone LMM741, Biolegend, San Diego, CA, USA), or with 1:20 PE control mouse IgG1 (BD Biosciences, San Jose, CA, USA). Then, cells were washed and stained for Vybrant DyeCycleTM (Life Technologies, Carlsbad, CA, USA) to discriminate cells that were alive, as described below. Sample fluorescence was measured by a seven-color MACSQuant Analyzer (Miltenyi Biotec), while data analysis was performed by using FlowJo software Version 8.8.6 (Tree Star, Ashland, OR, USA).

### Neutrophil viability

After an overnight treatment with the agonists indicated above, 10^5^ neutrophils were centrifuged at 300 × *g* for 5 min, medium removed and ultimately suspended in 100 μl HBSS buffer containing 10 nM Vybrant DyeCycleTM Violet stain (Life Technologies) and 5μM SYTOX AADvanced (Life Technologies). Cells were then put on ice for 30 min, protected from light. Cell viability was defined as the percentage of cells that were double negative for both stains (Vybrant/Sytox, respectively).

### Reverse transcription quantitative real-time PCR (RT-qPCR)

Total RNA was extracted from neutrophils after lysis by RNeasy Mini Kit (Qiagen, Venlo, Limburg, Netherlands), according to the manufacturer’s instructions. To completely remove any possible contaminating DNA, an on-column DNase digestion with the RNase-free DNase set (Qiagen) was performed during total RNA isolation. Purified total RNA was then reverse-transcribed into cDNA using Superscript III (Life Technologies) and random hexamer primers (Life Technologies) while qPCR was carried out using Fast SYBR® Green Master Mix (Life Technologies)^59^. Sequences of gene-specific primer pairs (Life Technologies) are available in the public database RTPrimerDB (www.rtprimerdb.org) under the following entry codes: GAPDH (3539), TNFα (3551), CXCL8 (3553), IL-6 (3545), PT-IL-6 (8687), IFNα_1–13_ (3541), IFNα_2_ (8955), IFN-β (3542), G-CSF (8615), SOCS3 (3828), TLR7 (8684), TLR8 (8685), IFIT1 (3540), MDA5 (3917), ISG15 (3547), LGP2 (3918) and OASL (3550). Data were calculated by Q-Gene software (http://www.gene-quantification.de/download.html) and expressed as mean normalized expression (MNE) units after GAPDH normalization.

### Immunoblots

Whole-cell proteins were recovered from protein-rich flow-through solutions after the first centrifugation step of the RNeasy Mini Kit (Qiagen) used for total RNA extraction, as previously described^8^. Proteins were then immunoblotted by standard procedures using the following antibodies: anti-IκBζ rabbit pAb (#9244) and anti-phospho-STAT3 (Tyr705) rabbit pAb (#9131) from Cell Signaling (Beverly, MA, USA); rabbit pAb anti-STAT3 (sc-482, Santa Cruz Biotechnology, Dallas, TX, USA); rabbit pAb anti-actin (A5060) from Sigma. Blotted proteins were detected and quantified by using the Odyssey infrared imaging system (LI-COR Biosciences, Lincoln, NE, USA). Densitometry values were calculated by subtracting, for each lane, the respective background levels.

### Chromatin Immunoprecipitation (ChIP) experiments

ChIP experiments were performed exactly as previously described^8^. Briefly, nuclear extracts from 5 × 10^6^ or 10^7^ neutrophils (for ChIP targeting, respectively, H3K27Ac or STAT1, IRF1, C/EBPβ and PolII) were immunoprecipitated with 1 μl anti-H3K27Ac (ab4729) (Abcam, Cambridge, United Kingdom), 25 μl anti-STAT1 (sc-346), 25 μl anti-IRF1 (sc-497), 20 μl anti- C/EBPβ (sc-150), 20 μl anti-PolII (sc-899) (Santa Cruz Biotechnology). To establish the background levels of ChIP experiments, the precipitation signal was quantified also at the promoter of prolactin (*PRL*) since it is completely silent in myeloid cells. The coimmunoprecipitated material was subjected to qPCR analysis using the following promoter specific primers (purchased from Life Technologies): IL-6 forward: 5′-TAGCCTCAATGACGACCTAAG-3′; IL-6 reverse: 5′-GTGGGGCTGATTGGAAACCT-3′; IFIT1 forward: 5′-GGCAGCAATGGACTGATGTTC-3′; IFIT1 reverse: 5′-GGAAACCGAAAGGGGAAAGTG-3′; and PRL forward: 5′-AGGGAAACGAATGCCTGATT-3′; PRL reverse: 5′-GCAGGAAACACACTTCACCA-3′. Data from qPCR were expressed as percentage over input DNA and are displayed as means ± SEM.

### Statistical analysis

Data are expressed as mean ± SE. Statistical evaluation was performed using Student’s t test, 1-way ANOVA followed by Tukey’s *post hoc* test or 2-way ANOVA followed by Bonferroni’s *post hoc* test. Values of P < 0.05 were considered as statistically significant.

### Study approval

Human samples were obtained following informed written consent by both healthy donors and SLE patients. Clinical evaluation of SLE diseases activity was assessed by the SLEDAI^60^ at the moment of the venipuncture. As reported in Table 1, only patients with moderate or severe flare (e.g., SLEDAI higher than 5) were recruited for our analysis. All experimental protocols were approved by the Ethic Committee of the Azienda Ospedaliera Universitaria Integrata di Verona (Italy). The methods were carried out in accordance with the approved guidelines.

## Additional Information

**How to cite this article**: Zimmermann, M. *et al.* IFNα enhances the production of IL-6 by human neutrophils activated *via* TLR8. *Sci. Rep.* **6**, 19674; doi: 10.1038/srep19674 (2016).

## Figures and Tables

**Table 1:** Characteristics of SLE patients.

Patient code	Age	Ethnicity^a^	Gender	Disease state (SLEDAI)	Symbol^b^
SLE # **1**	36	C	F	6	Δ
SLE # **2**	68	C	F	8	ο
SLE # **3**	23	C	F	5	□
SLE # **4**	27	H	F	14	◊
SLE # **5**	48	C	F	12	∇
SLE # **6**	42	C	F	11	*

^a^H = Hispanic; C = Caucasian.

^b^Symbols refer to Fig. 7a.
